# Association between Residential Exposure to Air Pollution and Incident Coronary Heart Disease Is Not Mediated by Leukocyte Telomere Length: A UK Biobank Study

**DOI:** 10.3390/toxics11060489

**Published:** 2023-05-28

**Authors:** Chia-Ling Kuo, Rui Liu, Lucas da Cunha Godoy, Luke C. Pilling, Richard H. Fortinsky, Doug Brugge

**Affiliations:** 1 The Cato T. Laurencin Institute for Regenerative Engineering, University of Connecticut Health, Farmington, CT 06030, USA; 2 Department of Public Health Sciences, University of Connecticut Health, Farmington, CT 06032, USA; 3 UConn Center on Aging, University of Connecticut Health, Farmington, CT 06030, USA; 4 Department of Health Sciences, Sacred Heart University, Fairfield, CT 06825, USA; 5 Epidemiology and Public Health Group, Faculty of Health and Life Sciences, University of Exeter, Exeter EX1 2LU, UK

**Keywords:** PM_2.5_, PM_2.5_ absorbance, PM_10_, PM_2.5–10_, NO_2_, NO_x_

## Abstract

Higher air pollution exposure and shorter leukocyte telomere length (LTL) are both associated with increased risk of coronary heart disease (CHD), and share plausible mechanisms, including inflammation. LTL may serve as a biomarker of air pollution exposure and may be intervened with to reduce the risk of CHD. To the best of our knowledge, we are the first to test the mediation effect of LTL in the relationship between air pollution exposure and incident CHD. Using the UK Biobank (UKB) data (*n* = 317,601), we conducted a prospective study linking residential air pollution exposure (PM_2.5_, PM_10_, NO_2_, NO_x_) and LTL to incident CHD during a mean follow-up of 12.6 years. Cox proportional hazards models and generalized additive models with penalized spline functions were used to model the associations of pollutant concentrations and LTL with incident CHD. We found non-linear associations of air pollution exposure with LTL and CHD. Pollutant concentrations in the lower range were decreasingly associated with longer LTL and reduced risk of CHD. The associations between lower pollutant concentrations and reduced risk of CHD, however, were minimally mediated by LTL (<3%). Our findings suggest that air pollution influences CHD through pathways that do not involve LTL. Replication is needed with improved measurements of air pollution that more accurately assesses personal exposure.

## 1. Introduction

Ambient air pollution, including particulate matter (PM) and oxides of nitrogen (NO_2_ and NO_x_) have been consistently and, in the case of PM_2.5_, causally associated with cardiovascular disease, including coronary heart disease (CHD) [1,2,3]. Due largely to cardiovascular health outcomes, PM_2.5_ is one of the leading causes of morbidity and mortality globally [4,5]. However, the biological pathways and mechanisms by which PM drives health outcomes remain under investigation, with many pathways, including inflammation [6,7,8].

Telomeres are repetitive base pair sequences at the end of chromosomes [9] that shorten with age and lead to cell cycle arrest and apoptosis when reaching a critical point [10]. Senescent cells secrete high levels of inflammatory cytokines, cell cycle regulators, growth factors, and tissue remodeling factors [11], which can contribute to cardiovascular disease [12]. The association of shorter leukocyte telomere length (LTL) with CHD, likely causal, is consistently replicated by observational studies and confirmed by Mendelian randomization studies that are robust to reverse causation and confounding [13,14,15].

Air pollution shares plausible mechanisms underlying the association between shorter LTL and CHD, including oxidative stress [16], chronic inflammation [17], and endothelial cell senescence [18,19]. It seems plausible that exposure to air pollution accelerates telomere shortening, which would, in turn, increase the risk of CHD. LTL, therefore, may serve as a biomarker of air pollution exposure and a prognostic factor for CHD. However, the association of exposure to air pollution with telomere length remains inconclusive [20,21,22]. We hypothesized that increased air pollution exposure is associated with CHD, and shorter LTL partially mediates the association.

To test our hypothesis, we conducted a prospective study linking residential air pollution exposure to LTL and incident CHD. We tested the mediation effect of LTL in the association between air pollution exposure and incident CHD during a mean follow-up of 12.6 years in the UK Biobank (UKB) cohort [23,24]. To the best of our knowledge, this is the first large-scale population study to explore the possible role of telomere biology in the association between air pollution exposure and the risk of CHD. Evidence of a mediating role of telomere length would support monitoring LTL for the risk of CHD due to exposure to air pollution.

## 2. Materials and Methods

### 2.1. UK Biobank

Data were obtained from the UKB, a large volunteer cohort in the United Kingdom. Over 500,000 participants were recruited from 2006 to 2010 with ages between 40 and 70 [23,24]. About 95% of the cohort are of European descent. At recruitment (baseline), participants completed online questionnaires, tests, and verbal interviews. They also performed physical assessments and provided biological samples for future assays. Through linkages to external datasets and electronic health records, additional data are available, including residential air pollution estimates and longitudinal follow-ups of disease diagnoses and death. Additional details about the cohort are described elsewhere, e.g., [23,24] and the UKB website [25].

### 2.2. Inclusion and Exclusion Criteria

**Figure 1:**
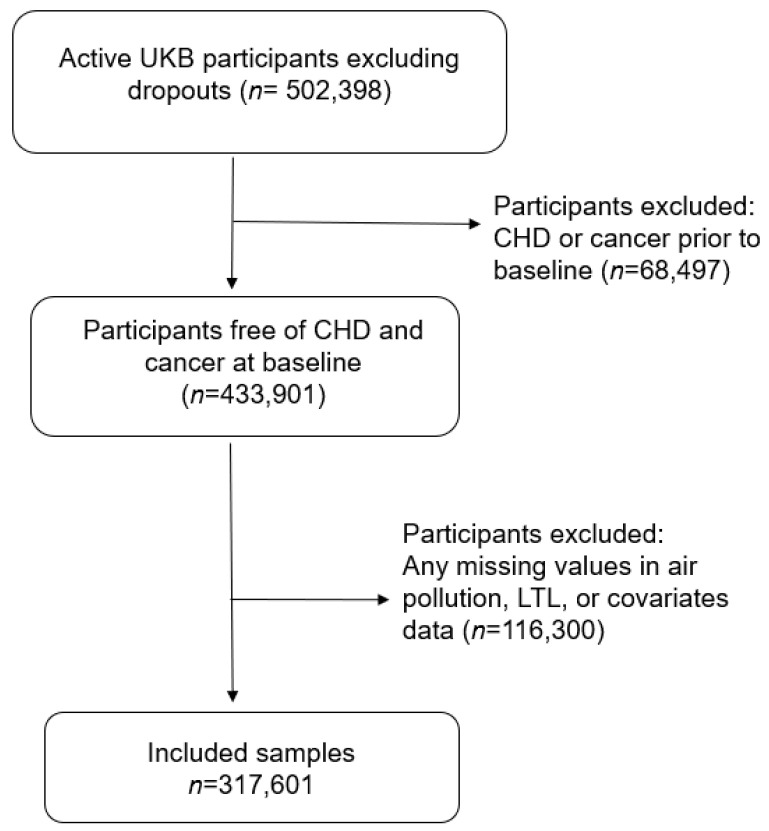
Sample selection flowchart.

We considered all the active UKB participants (*n* = 503,398) and excluded participants who had a diagnosis of CHD or cancer (excluding non-melanoma skin cancer) at or prior to baseline. Cancer is a potential confounder for the reported associations with longer LTL [14] and incident CHD [26]. We also excluded those with any missing values for air pollution, LTL, or covariates (*n* = 184,797) (Figure 1). Participants who did not have any CHD diagnosis in the records were assumed to have not developed CHD. A total of 317,601 study participants were included in our analysis.

### 2.3. Data

Air pollution, LTL, and covariates selected as potential confounders for associations with CHD were measured at baseline. Incident CHD during follow-up was the outcome of interest for associations with air pollution exposure and LTL to conduct a mediation analysis.

**Figure 2:**
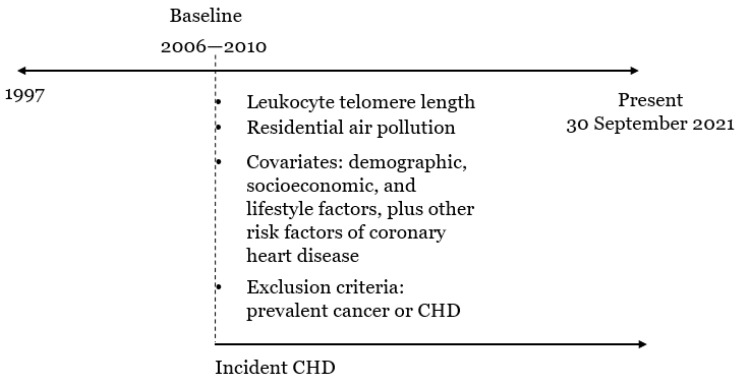
A timeline to illustrate the data collection.

The timeline in Figure 2 illustrates the data collection. Data were extracted using the field IDs in Table S1. As shown in Figure 2, the censoring date for incident coronary heart disease varied with data providers. It was set to 30 September 2021 in line with that of the Hospital Episode Statistics for England (HES), which was the main source used to confirm disease diagnoses [27]. Other data were collected at the recruitment/baseline of UKB.

#### 2.3.1. Residential Air Pollution

The air pollution monitoring data were collected in 2010 by the Small Area Health Statistics Unit [28] as part of the BioSHaRE-EU Environmental Determinants of Health Project [29]. Air pollution concentrations were modeled for nitrogen dioxide (NO_2_, µg/m^3^), nitrogen oxides (NO_x_; µg/m^3^), particulate matter of less than 10 um (PM_10_; µg/m^3^), PM_2.5_ (µg/m^3^), PM_2.5_ absorbance (per meter), and PM_2.5–10_ (µg/m^3^) using Land Use Regression (LUR) models (resolution 100 m × 100 m) provided by the European Study of Cohorts for Air Pollution Effects (ESCAPE, [30]) [31,32]. Notably, the ESCAPE estimates for particulates (PM_10_, PM_2.5_, PM_2.5_ absorbance, and PM coarse concentrations) more than 400 km away from the monitoring area, i.e., Greater London, might be invalid and were set to missing (*n* = 33,935) in the data UKB released. For sensitivity analysis, we analyzed data of PM_10_ and NO_2_ from previous years (prior to 2010) that were derived from EU-wide air pollution maps based on a LUR model (resolution 100 m × 100 m) [33]. The multi-year data were averaged, excluding data from 2010 to avoid potential batch effects.

#### 2.3.2. Leukocyte Telomere Length

Relative mean LTL (referred to simply as LTL hereafter) was measured from peripheral blood leukocytes as a T/S ratio using a multiplex qPCR technique by comparing the amount of the telomere amplification product (T) to that of a single-copy gene (S). LTL was used in this project after adjusting for the influence of technical parameters, as recommended by UKB [34].

#### 2.3.3. Disease Diagnoses

CHD diagnoses were confirmed based on ICD-10 codes (I20–I25) using the first occurrence data derived by UKB that linked multi-source data, including primary care, hospital inpatient, death register, and baseline self-reported medical condition data. First diagnosis dates were extracted for CHD cases to compare with the baseline assessment dates to determine a prevalent case at baseline or an incident case during follow-up. Throughout the study, CHD-free participants were censored at the last follow-up date of HES in England or the date of death, depending on which occurred first.

Cancer diagnoses are not included in the first occurrence data. Using cancer registry, hospital inpatient, and baseline self-reported medical condition data, participants diagnosed with any cancer (excluding non-melanoma skin cancer, ICD-10 C00–C97 excluding C44) at or prior to baseline were excluded from analysis.

#### 2.3.4. Covariates

Socio-demographic data included age, self-reported sex (male or female), ethnicity (grouped into White, Black, South Asian, and Other) and education (from none to college or university degree). The percentage of greenspace as a proportion of all land-use types was estimated at 1000 m buffers from the home address [35]. Lifestyle factors included body mass index (BMI), smoking status, alcohol intake frequency, and physical activity. Weight and height used to calculate BMI were physically measured at recruitment. Smoking status (never, former, or current) and alcohol intake frequency (never, special occasions only, one to three times a month, one or twice a week, three or four times a week, daily or almost daily) were assessed via online questionnaires. The physical activity group (low, moderate, or high) was self-reported and measured following the short International Physical Activity Questionnaire guideline [36].

### 2.4. Statistical Methods

Participant characteristics were descriptively summarized by the status of incident CHD. The groups with and without incident CHD were compared for categorical variables using chi-square tests and two-sided Wilcoxon rank-sum tests for continuous variables. Histograms were plotted to visualize the distributions of air pollutants. Air pollutant concentrations were correlated with each other using Spearman’s rank-based correlation.

Data of LTL and air pollutant concentrations were z-transformed according to the inverse normal transformation prior to the association analysis. Associations of air pollutants with LTL were examined using generalized additive models (GAMs). Cox proportional hazards models were used for LTL or air pollutants and incident CHD. The associations above were adjusted for covariates (age, sex, ethnicity, education, BMI, smoking status, alcohol intake frequency, physical activity group, and percent of greenspace percentage in 1000 m buffers), allowed to be nonlinear and modeled via penalized cubic spline functions (number of splines in the basis 10). The models above were fitted using the R functions: “cs” and “gam”, and “pspline” and “coxph”.

LTL and one air pollutant at a time were modeled jointly in a Cox proportional hazards model to explore the mediation effect of LTL in the association between the pollutant concentration and incident CHD. Additionally, we conducted a mediation analysis to test if the association between air pollution in the lower exposure range and protection of CHD was mediated by longer LTL. Due to a lack of tools to tackle the challenge of intensive computation, we conducted a mediation analysis using a linear regression model instead of a generalized additive model to model the association between air pollution and LTL, and a logistic regression model instead of a Cox regression model including a cubic spline function to model the association of air pollution and LTL with incident CHD. Specifically, we categorized the z-scores of each air pollutant into the ranges of (−Inf, −2], (−2, −1], (−1, −0.5], (−0.5, 0.5], (0.5, 1], (1, 2], and (2, Inf]. A linear regression model was used to model the mediation of LTL by comparing the mean LTL of a group with a lower range of exposure to air pollution ((−Inf, −2], (−2, −1], or (−1, −0.5]) to that of the reference group (−0.5, 0.5) with average exposure. A logistic regression model was used to model incident CHD including LTL and a level of pollutant concentration below the average. Both models were adjusted for covariates. The direct and indirect effects of a low pollutant concentration and proportion of effect mediated by LTL were reported by the status of incident CHD and on average. The mediation analysis was carried out using the R package “mediation” (a quasi-Bayesian approximation method to estimate confidence intervals) [37].

## 3. Results

### 3.1. Descriptive Analysis

The CHD incidence was 7.3% during a mean follow-up of 12.6 years (SD = 0.79), with the mean age at diagnosis 67.3 years (SD = 7.6). Older adults (median baseline age 61 years in CHD cases versus 56 years in CHD-free controls), men (10.2% versus 4.8% in women), and South Asian people (10.9% versus 7.3% in White people and 4.3% in Black people) were at higher risk of incident CHD (Table 1). Higher education and healthier lifestyles (lower BMI, no smoking, higher physical activity, and percentage of greenspace) were associated with a lower incidence of CHD (Table 1). In contrast, moderate alcohol consumption (1–3 times a month to 3–4 times a week to) was protective of incident CHD (Table 1).

Shorter LTL was observed in CHD cases (median 0.80 (T/S ratio)) than in CHD-free controls (median 0.83 (T/S ratio)) (Table 1). PM_2.5_, NO_2_, and NO_x_ in 2010 were higher at the residence of CHD cases than those at the residence of CHD-free controls (Table 1). The distributions of LTL and pollutant concentrations were somewhat right skewed (Figure S1). Air pollutant concentrations were positively correlated with each other (Figure S2). PM_2.5_, NO_2_, and NO_x_ in 2010 were highly correlated (Spearman *r* > 0.85) as were PM_2.5–10_ and PM_10_ in 2010 (Spearman *r* = 0.77). Interestingly, the correlation of PM_10_ concentrations between 2007 and 2010 was low (Spearman *r* = 0.43) in contrast with NO_2_ average concentrations in 2005–2007 and 2010 (Spearman *r* = 0.88).

### 3.2. Associations of Air Pollutants with Leukocyte Telomere Length

**Figure 3:**
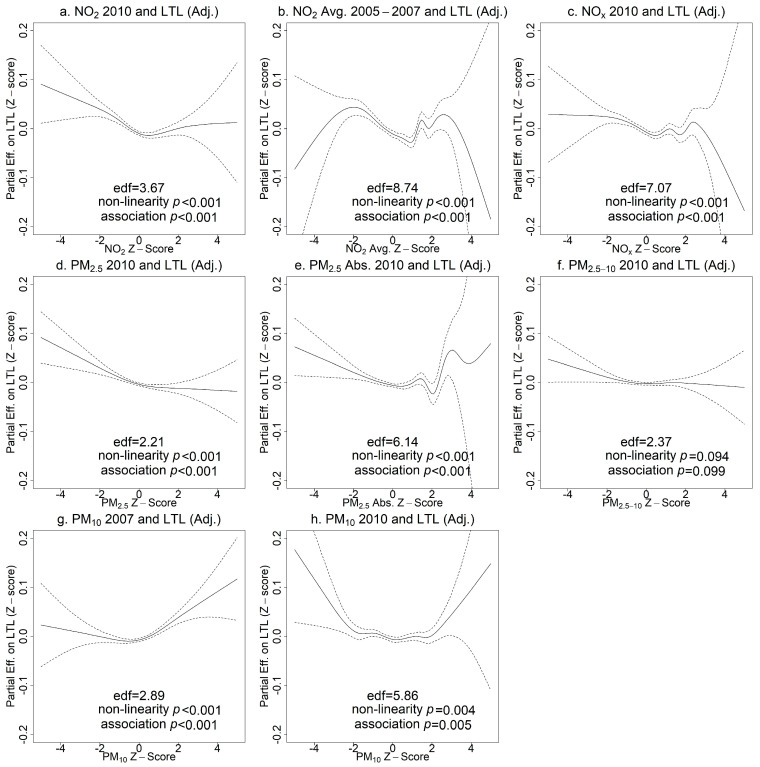
Generalized additive model (GAM) plots of partial effects of pollutant concentrations on leukocyte telomere length (LTL) adjusting for covariates (age, sex, ethnicity, education, BMI, smoking status, alcohol intake frequency, physical activity, percent of greenspace in 1000 m buffers). The tick marks on the *x*-axis are z-scores of the concentration of an air pollutant. The *y*-axis represents the partial effect of the concentration of an air pollutant. The areas between dashed lines indicate the 95% confidence intervals. (**a**) NO_2_ in 2010 and LTL; (**b**) NO_2_ average 2005–2007 and LTL; (**c**) NO_x_ in 2010 and LTL; (**d**) PM_2.5_ in 2010 and LTL; (**e**) PM_2.5_ absorbance in 2010 and LTL; (**f**) PM_2.5–10_ in 2010 and LTL; (**g**) PM_10_ in 2007 and LTL; (**h**) PM_10_ in 2010 and LTL.

The associations between pollutant concentrations and LTL showed significant non-linearity in unadjusted models (effective degrees of freedom (edf) >> 1 and non-linearity *p* < 0.05 in Figure S3) with wide confidence intervals at high and low concentrations due to smaller sample sizes. After adjusting for covariates, the non-linearity and the partial effect of the air pollutant on LTL were attenuated, but most non-linear associations remained except for PM_2.5–10_ in 2010 (*p* > 0.05). As shown in Figure 3, an upward trend on the left indicated that NO_2_, NO_x_, PM_2.5_, and PM_2.5_ absorbance concentrations in the lower range were associated with longer LTL. Higher concentrations of these air pollutants did not appear to be associated with LTL based on the majority of the data (z-scores between 0 and 2). The associations of extremely high pollutant concentrations (z-scores > 2) with LTL were uncertain, as reflected in the wide confidence intervals and substantial deviations from monotonic curves. Interestingly, PM_10_ showed a U-shaped relationship with LTL, but again with substantial uncertainty.

### 3.3. Associations of Leukocyte Telomere Length and Air Pollutants with Incident Coronary Heart Disease

**Figure 4:**
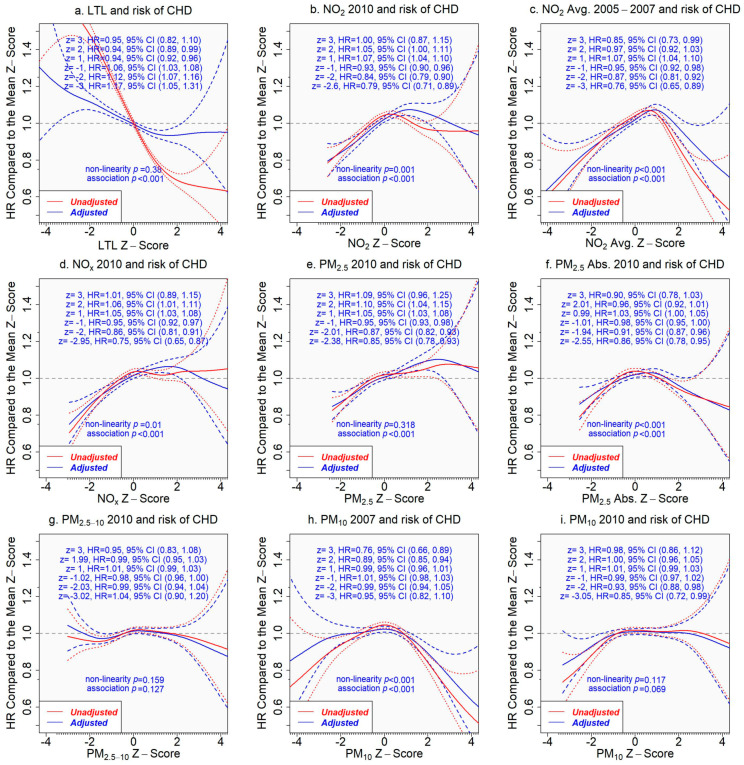
Associations of pollutant concentrations or leukocyte telomere length (LTL) with incident coronary heart disease (CHD), with or without adjustment for covariates. The tick marks on the *x*-axis are z-scores of leukocyte telomere length or the pollutant concentration. The *y*-axis represents the hazard ratio of incident coronary heart disease, comparing a given z-score to the mean z-score (=0) of the *x*-axis variable. The areas between the dashed lines indicate the 95% confidence intervals. Adjusted results are in blue versus the unadjusted results in red. (**a**) LTL and incident CHD; (**b**) NO_2_ in 2010 and incident CHD; (**c**) NO_2_ average 2005–2007 and incident CHD; (**d**) NO_x_ in 2010 and incident CHD; (**e**) PM_2.5_ in 2010 and incident CHD; (**f**) PM_2.5_ absorbance in 2010 and incident CHD; (**g**) PM_2.5–10_ in 2010 and incident CHD; (**h**) PM_10_ in 2007 and incident CHD; (**i**) PM_10_ in 2010 and incident CHD.

The association of LTL with incident CHD was significantly reduced after adjusting for covariates, and the nonlinearity was no longer statistically significant (*p* > 0.05) (Figure 4a). Assuming a linear relationship between LTL and incident CHD in a Cox proportional hazards model, the adjusted hazard ratio (HR) of incident CHD per SD increase in LTL was 0.95 (95% CI 0.94 to 0.96, *p* < 0.001). Without the linearity assumption, the adjusted HR comparing a given z-score to the mean z-score (=0) of LTL is presented in Figure 4. For example, the adjusted HR comparing z = 2 to z = 0 was 0.94 (95% CI 0.89 to 0.99) versus 1.12 (95% CI 1.07 to 1.16) comparing z = −2 to z = 0 (Figure 4a). The selected z-scores were the observed z-scores closest to −3, −2, −1, 1, 2, and 3 for LTL and for individual air pollutants.

In contrast, both unadjusted and adjusted associations of pollutant concentrations with incident CHD were similar (Figure 4). Pollutant concentrations in the lower range were positively associated with the risk of incident CHD (excluding PM_2.5–10_ and PM_10_). The risk of developing CHD increased when the pollutant concentration was above the average, but the risk dropped after the concentration reached a critical level, which varied with air pollutants, e.g., z-score 2 for NO_x_, 3 for PM_2.5_, and 1 for NO_2_ (Figure 4). However, the associations of extremely high pollutant concentrations with incident CHD came with great uncertainty.

### 3.4. Does LTL Mediate the Associations between Air Pollutants and Incident Heart Disease?

**Figure 5:**
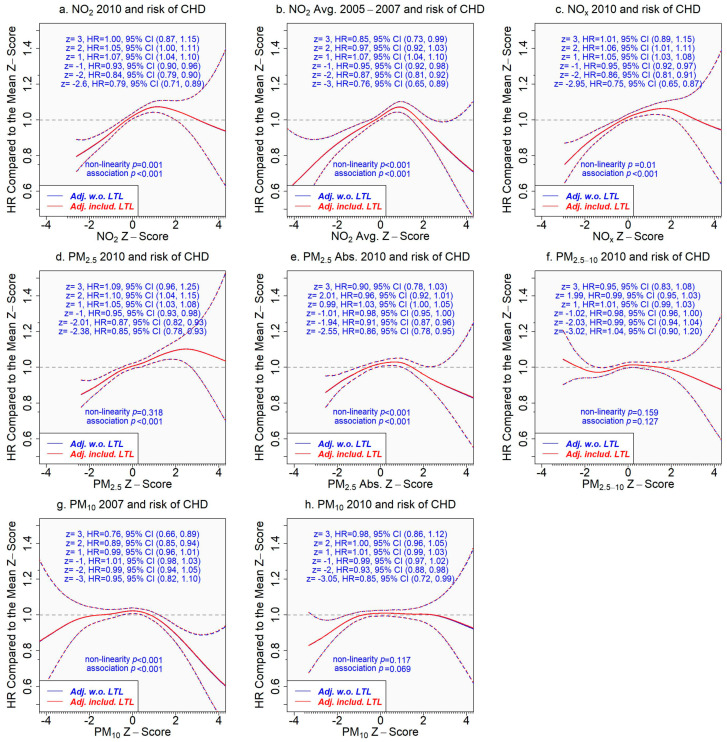
Association of the concentration of an air pollutant with incident coronary heart disease adjusting for covariates only or covariates plus leukocyte telomere length. The tick marks on the *x*-axis are z-scores of the concentration of an air pollutant. The *y*-axis represents the hazard ratio of incident coronary heart disease comparing a given z-score to the mean z-score (=0) of the *x*-axis variable. The areas between dash lines indicate the 95% confidence intervals. Results adjusting for covariates only are in blue versus the results in red adjusting for covariates and leukocyte telomere length (which assumed a linear relationship in the model due to insignificant non-linearity *p* = 0.380 (Figure 4)). (**a**) NO_2_ in 2010 and incident CHD; (**b**) NO_2_ average 2005–2007 and incident CHD; (**c**) NO_x_ in 2010 and incident CHD; (**d**) PM_2.5_ in 2010 and incident CHD; (**e**) PM_2.5_ absorbance in 2010 and incident CHD; (**f**) PM_2.5–10_ in 2010 and incident CHD; (**g**) PM_10_ in 2007 and incident CHD; (**h**) PM_10_ in 2010 and incident CHD.

If the associations of air pollutants with incident CHD are mediated by LTL, we would expect that adjusting the associations for LTL would attenuate them significantly. The mediating role of LTL, however, was not supported by our results. We found similar hazard ratios for incident CHD when comparing a z-score to the mean z-score (=0) of the concentration of an air pollutant from the models adjusting for covariates only and for covariates plus LTL (assuming a linear relationship with incident CHD, since non-linearity was not significant, *p* = 0.380 (Figure 4)) (Figure 5).

Next, we considered the air pollutants that showed inverse associations with LTL and CHD at the lower range of their concentrations, i.e., NO_2_, NO_x_, PM_2.5_, and PM_2.5_ absorbance. We conducted an analysis to estimate the mediation effect of LTL on the association between pollutant concentrations in the lower range and the development of CHD. The proportion of effect on CHD mediated by LTL comparing a lower range of pollutant concentration to the range centered at the mean was less than 3% across the air pollutants (Tables S2–S6), providing evidence against the mediating role of LTL in the association.

## 4. Discussion

While we did not find consistent and monotonic associations of air pollutants with CHD across the entire concentration range of pollutants, we did find associations in the lower concentration range for several pollutants. Pollutant concentrations in the lower range were decreasingly associated with longer LTL and a lower risk of CHD. Additionally, longer LTL was associated with a lower risk of CHD, which is consistent with the prior literature [13,14,15].

The primary innovation of our analysis was to test whether LTL mediated the association between air pollution exposure and incident CHD. There is considerable evidence that air pollution drives adverse cardiovascular health outcomes through inflammation [6,7]. To our knowledge, there has been little research on the possible role of telomere length as a biological pathway from air pollution exposure to CHD. This pathway is plausible since LTL has been linked to inflammation [38] and air pollution is also associated with inflammatory responses [6].

The secondary innovation of our analysis was to adopt non-linear modeling that allows the exposure–outcome relationship to vary with the exposure level. We found monotone associations of air pollution exposure with LTL and CHD only in the lower range of pollutant concentrations. In contrast, the associations in the higher range of pollutant concentration were inconclusive due to greater uncertainty, which will require further investigation. Contradictory to our findings, one study [22] showed no significant association between air pollution exposure and LTL using the same data source. The inconsistency may be partly explained by modeling differences. Notably, they assessed non-linearity via a quadratic term and found no strong evidence across pollutants.

The evidence from our mediation analysis does not support the hypothesis that LTL is on the biological pathway between air pollution exposure and CHD. LTL and exposure to air pollution were independently associated with CHD across the whole range of pollutant concentration. The mediated effects of LTL in the associations between pollutant concentrations in the lower range (z scores in (−Inf, −2], (−2, −1], or (−1, −0.5] versus z scores in (−0.5, 0.5]) and CHD were all less than 3% across the pollutants we analyzed. Thus, we would suggest that it is more likely that air pollution in the lower range influences CHD through pathways that do not involve LTL. Future studies may address both mediation and moderation effects of telomere length on acute cardiovascular events in addition to CHD.

Our analysis has several strengths. First, the prospective study design ensured the temporality of the effects of air pollution exposure and LTL on incident CHD. Second, we modeled the whole range of a form of exposure (e.g., exposure to air pollution) to determine the effect on an outcome (e.g., LTL) without a presumption of a linear relationship. Third, the attrition rate was low, as participants were followed up for health outcomes through electronic linkages. Fourth, the air pollution models have good accuracy for estimating ambient concentrations at residences, albeit more so for PM_2.5_ than PM_10_ or NO_2_. Fifth, multiple air pollutants were included, which is a priority in present day environmental epidemiology research. Finally, the sample size was large, providing substantial statistical power, and the LTL data were collected through rigorous methods with high levels of quality control.

Despite these considerable strengths, there are some limitations to the source data we used. First, telomeres from cardiac tissues may be more relevant to this study than peripheral blood leukocytes, but the correlations between telomere length among different tissues are generally positive [39]. Moreover, data on LTL over time are not available for study of the mediating effect of telomere attrition in the association between air pollution exposure and incident CHD. Second, ambient concentrations of pollutants in the homes of study participants are widely used; however, this estimation of exposure likely contains errors because of self-reported addresses, errors from matching in geographic information systems, and differences indoors and the movement of participants to other locations. We have less confidence in the PM_10_ data because they varied considerably by year. Exposure misclassification would likely bias effect estimates toward the null [40]. Third, we did not consider other potential mediators, likely also related to inflammation but independent of LTL to CHD. Although we included many possible confounders as co-variates, there remains the possibility our analysis does not meet the assumptions of no unmeasured confounding for the mediation analysis. However, the associations of air pollutants with incident CHD minimally changed after adjusting for LTL, suggesting no mediating effect of LTL. Fourth, variables such as the UKB baseline assessment center and Townsend deprivation index were measured by area, and they more or less overlapped with the ambient pollutant concentrations, so they were not adjusted in statistical models. However, we used individual data of education, which can serve as a proxy for socio-economic status. Fifth, the sample size was significantly reduced for extreme telomere length and pollutant concentrations, which come with greater uncertainty in estimation and restrict additional analyses to separate populations with differential vulnerability to air pollution exposure, e.g., women versus men and older adults versus younger adults [41]. Finally, generalizability is reduced by the limited racial/ethnic composition of the study population and its geographic location in the UK.

## 5. Conclusions

In a large, well-defined health cohort, we confirmed the association between shorter LTL and higher risk of CHD. Our analysis showed non-linear relationships of exposure to air pollution with LTL and CHD. Specifically, increased exposure to air pollution in the lower range was associated with shorter LTL and higher risk of CHD. The association between air pollution exposure and CHD, however, was not mediated by LTL. Our findings suggest that it is more likely that air pollution in the lower range influences CHD through pathways that do not involve LTL. An area for future research would be to improve exposure assignment of air pollution to get closer to actual personal exposure.

## Figures and Tables

**Table 1:** Participant characteristics of the included samples (*n* = 317,601).

Characteristics	Incident CHD (*n* = 23,089)	No Incident CHD (*n* = 294,512)	*p*-Value
Baseline age, years (median (first quartile, third quartile))	61 (56, 65)	56 (49, 62)	<0.001
Sex (%)			<0.001
Male	14,868 (10.2%)	131,231 (89.8%)	
Female	8221 (4.8%)	163,281 (95.2%)	
Ethnicity (%)			<0.001
White	21,859 (7.3%)	278,118 (92.7%)	
Black	229 (4.3%)	5144 (95.7%)	
South Asian	690 (10.9%)	5614 (89.1%)	
Other	311 (5.2%)	5636 (94.8%)	
Education (%)			<0.001
None	5448 (11.7%)	41,167 (88.3%)	
CSEs or equivalent	771 (6.1%)	11,930 (93.9%)	
O levels/GCSEs or equivalent	2948 (7.0%)	39,262 (93.0%)	
A/AS levels/NVQ/HND/HNC	4273 (7.2%)	55,293 (92.8%)	
Other professional qualifications	3523 (7.4%)	44,089 (92.6%)	
College or university degree	6126 (5.6%)	102,771 (94.4%)	
BMI, kg/m^2^ (mean ± SD)	27.83 (25.22, 31)	26.43 (23.91, 29.45)	
Smoking status (%)			<0.001
Never	10,715 (6.0%)	167,879 (94.0%)	
Previous	9205 (8.6%)	98,448 (91.4%)	
Current	3169 (10.1%)	28,185 (89.9%)	
Alcohol intake frequency (%)			<0.001
Never	2141 (9.4%)	20,746 (90.6%)	
Special occasions only	2714 (8.0%)	31,224 (92.0%)	
1–3 times a month	2379 (6.8%)	32,646 (93.2%)	
1–2 times a week	5665 (6.9%)	76,576 (93.1%)	
3–4 times a week	5021 (6.6%)	71,434 (93.4%)	
Daily or almost daily	5169 (7.7%)	61,886 (92.3%)	
IPAQ activity group (%)			<0.001
Low	3729 (8.1%)	42,379 (91.9%)	
Moderate	10,162 (7.3%)	129,905 (92.7%)	
High	9198 (7.0%)	122,228 (93.0%)	<0.001
Greenspace percentage (buffer 1000 m)	42.22 (28.11, 59.68)	41.89 (27.4, 60.59)	0.161
Telomere length (T/S ratio), adjusting for technical parameters	0.80 (0.73, 0.88)	0.83 (0.75, 0.91)	<0.001
NO_2_ in 2010 (µg/m^3^)	26.24 (21.58, 31.22)	26.05 (21.22, 31.23)	<0.001
NO_2_ in 2005 (µg/m^3^)	28.89 (23.55, 35.22)	28.67 (23.19, 35.37)	0.028
NO_2_ in 2006 (µg/m^3^)	28.28 (23.15, 33.79)	28.06 (22.81, 33.77)	0.002
NO_2_ in 2007 (µg/m^3^)	29.43 (24.14, 35.49)	29.29 (23.75, 35.65)	0.099
NO_2_ avg. (2005, 2006, 2007) (µg/m^3^)	28.86 (23.65, 34.81)	28.71 (23.28, 34.9)	0.023
NO_x_ in 2010 (µg/m^3^)	42.39 (34.61, 50.85)	42 (33.93, 50.64)	<0.001
PM_2.5_ in 2010 (µg/m^3^)	9.95 (9.32, 10.58)	9.91 (9.27, 10.55)	<0.001
PM_2.5_ absorbance in 2010 (per meter)	1.13 (1.00, 1.3)	1.13 (0.99, 1.31)	0.386
PM_2.5–10_ in 2010 (µg/m^3^)	6.11 (5.85, 6.63)	6.11 (5.84, 6.63)	0.169
PM_10_ in 2007 (µg/m^3^)	21.96 (20.59, 23.7)	22.00 (20.56, 23.89)	0.002
PM_10_ in 2010 (µg/m^3^)	16.03 (15.27, 16.98)	16.02 (15.23, 16.99)	0.038

## Data Availability

The UKB data are available upon approved request.

## Supplementary Materials

The following supporting information can be downloaded at: https://www.mdpi.com/article/10.3390/toxics11060489/s1, Table S1: UK Biobank field IDs to extract data; Table S2: Casual mediation analysis to estimate average causal mediation effects (ACMEs) through leukocyte telomere length and average direct effects (ADE) of NO_2_ concentrations in 2010 in the lower range vs. the average on the risk of incident coronary heart disease; Table S3: Casual mediation analysis to estimate average causal mediation effects (ACMEs) through leukocyte telomere length and average direct effects (ADE) of NO_2_ concentration averages from 2005 to 2007 in the lower range vs. the average on the risk of incident coronary heart disease; Table S4: Casual mediation analysis to estimate average causal mediation effects (ACMEs) through leukocyte telomere length and average direct effects (ADE) of NO_x_ concentrations in 2010 in the lower range vs. the average on the risk of incident coronary heart disease; Table S5: Casual mediation analysis to estimate average causal mediation effects (ACMEs) through leukocyte telomere length and average direct effects (ADE) of PM_2.5_ concentrations in 2010 in the lower range vs. the average on the risk of incident coronary heart disease; Table S6: Casual mediation analysis to estimate average causal mediation effects (ACMEs) through leukocyte telomere length and average direct effects (ADE) of PM_2.5_ absorbance concentrations in 2010 in the lower range vs. the average on the risk of incident coronary heart disease; Figure S1: Histograms of leukocyte telomere length and air pollutants; Figure S2: Spearman correlations between pollutant concentrations; Figure S3: Generalized additive model (GAM) plots of the partial effects of air pollutants on leukocyte telomere length, with no adjustment for covariates. The tick marks on the *x*-axis are z-scores of an air pollutant. The *y*-axis represents the partial effect of each air pollutant. The areas between dash lines indicate the 95% confidence intervals. (a) NO_2_ 2010 and LTL; (b) NO_2_ average 2005–2007 and LTL; (c) NO_x_ 2010 and LTL; (d) PM_2.5_ 2010 and LTL; (e) PM_2.5_ absorbance and LTL; (f) PM_2.5–10_ 2010 and LTL; (g) PM_10_ 2007 and LTL; (h) PM_10_ 2010 and LTL.
