# Supplementary material for: Association between Residential Exposure to Air Pollution and Incident Coronary Heart Disease Is Not Mediated by Leukocyte Telomere Length: A UK Biobank Study
Source: Toxics. 2023 May 28;11(6):489. doi: 10.3390/toxics11060489 (PMC10301073; doi:10.3390/toxics11060489)
Supplement: Supplementary file 1 [file toxics-11-00489-s001.zip › toxics-2358972-supplementary.pdf]

Supplementary Materials

Table S1. UK Biobank field IDs to extract data.

Field ID	Description
22191	Adjusted T/S ratio
24016	Nitrogen dioxide air pollution; 2005
24017	Nitrogen dioxide air pollution; 2006
24018	Nitrogen dioxide air pollution; 2007
24003	Nitrogen dioxide air pollution; 2010
24004	Nitrogen oxides air pollution; 2010
24019	Particulate matter air pollution (pm10); 2007
24005	Particulate matter air pollution (pm10); 2010
24007	Particulate matter air pollution (pm2.5) absorbance; 2010
24006	Particulate matter air pollution (pm2.5); 2010
24008	Particulate matter air pollution 2.5-10um; 2010
24500	Greenspace percentage, buffer 1000m
131296	Date I20 first reported (angina pectoris)
131298	Date I21 first reported (acute myocardial infarction)
131300	Date I22 first reported (subsequent myocardial infarction)
131302	Date I23 first reported (certain current complications following acute myocardial infarction)
131304	Date I24 first reported (other acute ischemic heart diseases)
131306	Date I25 first reported (chronic ischemic heart disease)
33	Date of birth
53	Date of attending assessment centre
31	Sex
21000	Ethnic background
6138	Qualifications
20116	Smoking status
22032	IPAQ activity group
1558	Alcohol intake frequency

Table S2. Casual mediation analysis to estimate average causal mediation effects (ACMEs) through leukocyte telomere length and average direct effects (ADE) of NO_2 concentrations in 2010 in the lower range vs. the average on the risk of incident coronary heart disease.

**NO_2 (-1,-0.5] vs. (-0.5, 0.5]**	**Estimate**	**95% CI Lower**	**95% CI Upper**	**P-Value**
ACME (-0.5, 0.5]	0.000	0.000	0.000	<2e-16
ACME (-1, 0.5]	0.000	0.000	0.000	<2e-16
ADE (-0.5, 0.5]	-0.004	-0.007	0.000	0.002
ADE (-1, 0.5]	-0.004	-0.007	0.000	0.002
Total Effect	-0.004	-0.007	0.000	0.002
Prop. Mediated (-0.5, 0.5]	0.016	0.006	0.050	0.002
Prop. Mediated (-1, 0.5]	0.015	0.005	0.050	0.002
**ACME (average)**	0.000	0.000	0.000	<2e-16
**ADE (average)**	-0.004	-0.007	0.000	0.002
**Prop. Mediated (average)**	0.015	0.006	0.050	0.002
**NO_2 (-2,-1] vs. (-0.5, 0.5]**				
ACME (-0.5, 0.5]	0.000	0.000	0.000	<2e-16
ACME (-2, -1]	0.000	0.000	0.000	<2e-16
ADE (-0.5, 0.5]	-0.009	-0.012	-0.010	<2e-16
ADE (-2, -1]	-0.009	-0.012	-0.010	<2e-16
Total Effect	-0.009	-0.012	-0.010	<2e-16
Prop. Mediated (-0.5, 0.5]	0.014	0.008	0.030	<2e-16
Prop. Mediated (-2, -1]	0.013	0.007	0.030	<2e-16
**ACME (average)**	0.000	0.000	0.000	<2e-16
**ADE (average)**	-0.009	-0.012	-0.010	<2e-16
**Prop. Mediated (average)**	0.014	0.007	0.030	<2e-16
**NO_2 (-Inf,-2] vs. (-0.5, 0.5]**				
ACME (-0.5, 0.5]	0.000	0.000	0.000	<2e-16
ACME (-Inf, -2]	0.000	0.000	0.000	<2e-16
ADE (-0.5, 0.5]	-0.016	-0.022	-0.010	<2e-16
ADE (-Inf, -2]	-0.016	-0.022	-0.010	<2e-16
Total Effect	-0.016	-0.022	-0.010	<2e-16
Prop. Mediated (-0.5, 0.5]	0.009	0.004	0.020	<2e-16
Prop. Mediated (-Inf, -2]	0.008	0.003	0.020	<2e-16
**ACME (average)**	0.000	0.000	0.000	<2e-16
**ADE (average)**	-0.016	-0.022	-0.010	<2e-16
**Prop. Mediated (average)**	0.008	0.003	0.020	<2e-16

Table S3. Casual mediation analysis to estimate average causal mediation effects (ACMEs) through leukocyte telomere length and average direct effects (ADE) of NO_2 concentration averages from 2005 to 2007 in the lower range vs. the average on the risk of incident coronary heart disease.

**NO_2 Avg. (-1,-0.5] vs. (-0.5, 0.5]**	**Estimate**	**95% CI Lower**	**95% CI Upper**	**P-Value**
ACME (-0.5, 0.5]	0.000	0.000	0.000	<2e-16
ACME (-1, 0.5]	0.000	0.000	0.000	<2e-16
ADE (-0.5, 0.5]	-0.003	-0.005	0.000	0.094
ADE (-1, 0.5]	-0.003	-0.005	0.000	0.094
Total Effect	-0.003	-0.006	0.000	0.09
Prop. Mediated (-0.5, 0.5]	0.030	-0.156	0.190	0.09
Prop. Mediated (-1, 0.5]	0.029	-0.157	0.190	0.09
**ACME (average)**	0.000	0.000	0.000	<2e-16
**ADE (average)**	-0.003	-0.005	0.000	0.094
**Prop. Mediated (average)**	0.030	-0.156	0.190	0.09
**NO_2 Avg. (-2,-1] vs. (-0.5, 0.5]**				
ACME (-0.5, 0.5]	0.000	0.000	0.000	<2e-16
ACME (-2, -1]	0.000	0.000	0.000	<2e-16
ADE (-0.5, 0.5]	-0.007	-0.011	0.000	<2e-16
ADE (-2, -1]	-0.007	-0.011	0.000	<2e-16
Total Effect	-0.007	-0.011	0.000	<2e-16
Prop. Mediated (-0.5, 0.5]	0.016	0.007	0.030	<2e-16
Prop. Mediated (-2, -1]	0.015	0.007	0.030	<2e-16
**ACME (average)**	0.000	0.000	0.000	<2e-16
**ADE (average)**	-0.007	-0.011	0.000	<2e-16
**Prop. Mediated (average)**	0.015	0.007	0.030	<2e-16
**NO_2 Avg. (-Inf,-2] vs. (-0.5, 0.5]**				
ACME (-0.5, 0.5]	0.000	0.000	0.000	<2e-16
ACME (-Inf, -2]	0.000	0.000	0.000	<2e-16
ADE (-0.5, 0.5]	-0.011	-0.018	0.000	0.002
ADE (-Inf, -2]	-0.011	-0.018	0.000	0.002
Total Effect	-0.012	-0.018	0.000	0.002
Prop. Mediated (-0.5, 0.5]	0.012	0.005	0.030	0.002
Prop. Mediated (-Inf, -2]	0.010	0.004	0.030	0.002
**ACME (average)**	0.000	0.000	0.000	<2e-16
**ADE (average)**	-0.011	-0.018	0.000	0.002
**Prop. Mediated (average)**	0.011	0.004	0.030	0.002

Table S4. Casual mediation analysis to estimate average causal mediation effects (ACMEs) through leukocyte telomere length and average direct effects (ADE) of NO_x concentrations in 2010 in the lower range vs. the average on the risk of incident coronary heart disease.

**NO_x (-1,-0.5] vs. (-0.5, 0.5]**	**Estimate**	**95% CI Lower**	**95% CI Upper**	**P-Value**
ACME (-0.5, 0.5]	0.000	0.000	0.000	<2e-16
ACME (-1, 0.5]	0.000	0.000	0.000	<2e-16
ADE (-0.5, 0.5]	-0.004	-0.006	0.000	0.02
ADE (-1, 0.5]	-0.004	-0.006	0.000	0.02
Total Effect	-0.004	-0.006	0.000	0.02
Prop. Mediated (-0.5, 0.5]	0.017	0.005	0.070	0.02
Prop. Mediated (-1, 0.5]	0.016	0.005	0.070	0.02
**ACME (average)**	0.000	0.000	0.000	<2e-16
**ADE (average)**	-0.004	-0.006	0.000	0.02
**Prop. Mediated (average)**	0.016	0.005	0.070	0.02
**NO_x (-2,-1] vs. (-0.5, 0.5]**				
ACME (-0.5, 0.5]	0.000	0.000	0.000	<2e-16
ACME (-2, -1]	0.000	0.000	0.000	<2e-16
ADE (-0.5, 0.5]	-0.007	-0.009	0.000	<2e-16
ADE (-2, -1]	-0.007	-0.009	0.000	<2e-16
Total Effect	-0.007	-0.009	0.000	<2e-16
Prop. Mediated (-0.5, 0.5]	0.012	0.005	0.030	<2e-16
Prop. Mediated (-2, -1]	0.011	0.004	0.020	<2e-16
**ACME (average)**	0.000	0.000	0.000	<2e-16
**ADE (average)**	-0.007	-0.009	0.000	<2e-16
**Prop. Mediated (average)**	0.012	0.004	0.020	<2e-16
**NO_x (-Inf,-2] vs. (-0.5, 0.5]**				
ACME (-0.5, 0.5]	0.000	0.000	0.000	0.004
ACME (-Inf, -2]	0.000	0.000	0.000	0.004
ADE (-0.5, 0.5]	-0.015	-0.021	-0.010	<2e-16
ADE (-Inf, -2]	-0.015	-0.021	-0.010	<2e-16
Total Effect	-0.015	-0.021	-0.010	<2e-16
Prop. Mediated (-0.5, 0.5]	0.008	0.003	0.020	0.004
Prop. Mediated (-Inf, -2]	0.007	0.002	0.010	0.004
**ACME (average)**	0.000	0.000	0.000	0.004
**ADE (average)**	-0.015	-0.021	-0.010	<2e-16
**Prop. Mediated (average)**	0.007	0.002	0.020	0.004

Table S5. Casual mediation analysis to estimate average causal mediation effects (ACMEs) through leukocyte telomere length and average direct effects (ADE) of $PM_{2.5}$ concentrations in 2010 in the lower range vs. the average on the risk of incident coronary heart disease.

**$PM_{2.5}$ (-1,-0.5] vs. (-0.5, 0.5]**	**Estimate**	**95% CI Lower**	**95% CI Upper**	**P-Value**
ACME (-0.5, 0.5]	0.000	0.000	0.000	<2e-16
ACME (-1, 0.5]	0.000	0.000	0.000	<2e-16
ADE (-0.5, 0.5]	-0.003	-0.006	0.000	0.032
ADE (-1, 0.5]	-0.003	-0.006	0.000	0.032
Total Effect	-0.003	-0.006	0.000	0.028
Prop. Mediated (-0.5, 0.5]	0.021	0.006	0.120	0.028
Prop. Mediated (-1, 0.5]	0.020	0.005	0.120	0.028
**ACME (average)**	0.000	0.000	0.000	<2e-16
**ADE (average)**	-0.003	-0.006	0.000	0.032
**Prop. Mediated (average)**	0.020	0.005	0.120	0.028
**$PM_{2.5}$ (-2,-1] vs. (-0.5, 0.5]**				
ACME (-0.5, 0.5]	0.000	0.000	0.000	0.004
ACME (-2, -1]	0.000	0.000	0.000	0.004
ADE (-0.5, 0.5]	-0.007	-0.010	0.000	<2e-16
ADE (-2, -1]	-0.007	-0.010	0.000	<2e-16
Total Effect	-0.007	-0.011	0.000	<2e-16
Prop. Mediated (-0.5, 0.5]	0.009	0.003	0.020	0.004
Prop. Mediated (-2, -1]	0.009	0.003	0.020	0.004
**ACME (average)**	0.000	0.000	0.000	0.004
**ADE (average)**	-0.007	-0.010	0.000	<2e-16
**Prop. Mediated (average)**	0.009	0.003	0.020	0.004
**$PM_{2.5}$ (-Inf,-2] vs. (-0.5, 0.5]**				
ACME (-0.5, 0.5]	0.000	0.000	0.000	<2e-16
ACME (-Inf, -2]	0.000	0.000	0.000	<2e-16
ADE (-0.5, 0.5]	-0.012	-0.017	-0.010	<2e-16
ADE (-Inf, -2]	-0.012	-0.017	-0.010	<2e-16
Total Effect	-0.012	-0.017	-0.010	<2e-16
Prop. Mediated (-0.5, 0.5]	0.014	0.007	0.030	<2e-16
Prop. Mediated (-Inf, -2]	0.012	0.006	0.030	<2e-16
**ACME (average)**	0.000	0.000	0.000	<2e-16
**ADE (average)**	-0.012	-0.017	-0.010	<2e-16
**Prop. Mediated (average)**	0.013	0.006	0.030	<2e-16

Table S6. Casual mediation analysis to estimate average causal mediation effects (ACMEs) through leukocyte telomere length and average direct effects (ADE) of $PM_{2.5}$ absorbance concentrations in 2010 in the lower range vs. the average on the risk of incident coronary heart disease.

**$PM_{2.5}$ Abs. (-1,-0.5] vs. (-0.5, 0.5]**	**Estimate**	**95% CI Lower**	**95% CI Upper**	**P-Value**
ACME (-0.5, 0.5]	0.000	0.000	0.000	0.408
ACME (-1, 0.5]	0.000	0.000	0.000	0.408
ADE (-0.5, 0.5]	-0.004	-0.006	0.000	0.012
ADE (-1, 0.5]	-0.004	-0.006	0.000	0.012
Total Effect	-0.004	-0.006	0.000	0.012
Prop. Mediated (-0.5, 0.5]	0.004	-0.006	0.020	0.412
Prop. Mediated (-1, 0.5]	0.004	-0.006	0.020	0.412
**ACME (average)**	0.000	0.000	0.000	0.408
**ADE (average)**	-0.004	-0.006	0.000	0.012
**Prop. Mediated (average)**	0.004	-0.006	0.020	0.412
**$PM_{2.5}$ Abs. (-2,-1] vs. (-0.5, 0.5]**				
ACME (-0.5, 0.5]	0.000	0.000	0.000	<2e-16
ACME (-2, -1]	0.000	0.000	0.000	<2e-16
ADE (-0.5, 0.5]	-0.006	-0.009	0.000	<2e-16
ADE (-2, -1]	-0.006	-0.009	0.000	<2e-16
Total Effect	-0.006	-0.009	0.000	<2e-16
Prop. Mediated (-0.5, 0.5]	0.012	0.005	0.030	<2e-16
Prop. Mediated (-2, -1]	0.011	0.004	0.030	<2e-16
**ACME (average)**	0.000	0.000	0.000	<2e-16
**ADE (average)**	-0.006	-0.009	0.000	<2e-16
**Prop. Mediated (average)**	0.011	0.004	0.030	<2e-16
**$PM_{2.5}$ Abs. (-Inf,-2] vs. (-0.5, 0.5]**				
ACME (-0.5, 0.5]	0.000	0.000	0.000	0.17
ACME (-Inf, -2]	0.000	0.000	0.000	0.17
ADE (-0.5, 0.5]	-0.011	-0.017	0.000	<2e-16
ADE (-Inf, -2]	-0.011	-0.017	0.000	<2e-16
Total Effect	-0.011	-0.017	0.000	<2e-16
Prop. Mediated (-0.5, 0.5]	0.006	-0.003	0.020	0.17
Prop. Mediated (-Inf, -2]	0.005	-0.003	0.020	0.17
**ACME (average)**	0.000	0.000	0.000	0.17
**ADE (average)**	-0.011	-0.017	0.000	<2e-16
**Prop. Mediated (average)**	0.005	-0.003	0.020	0.17

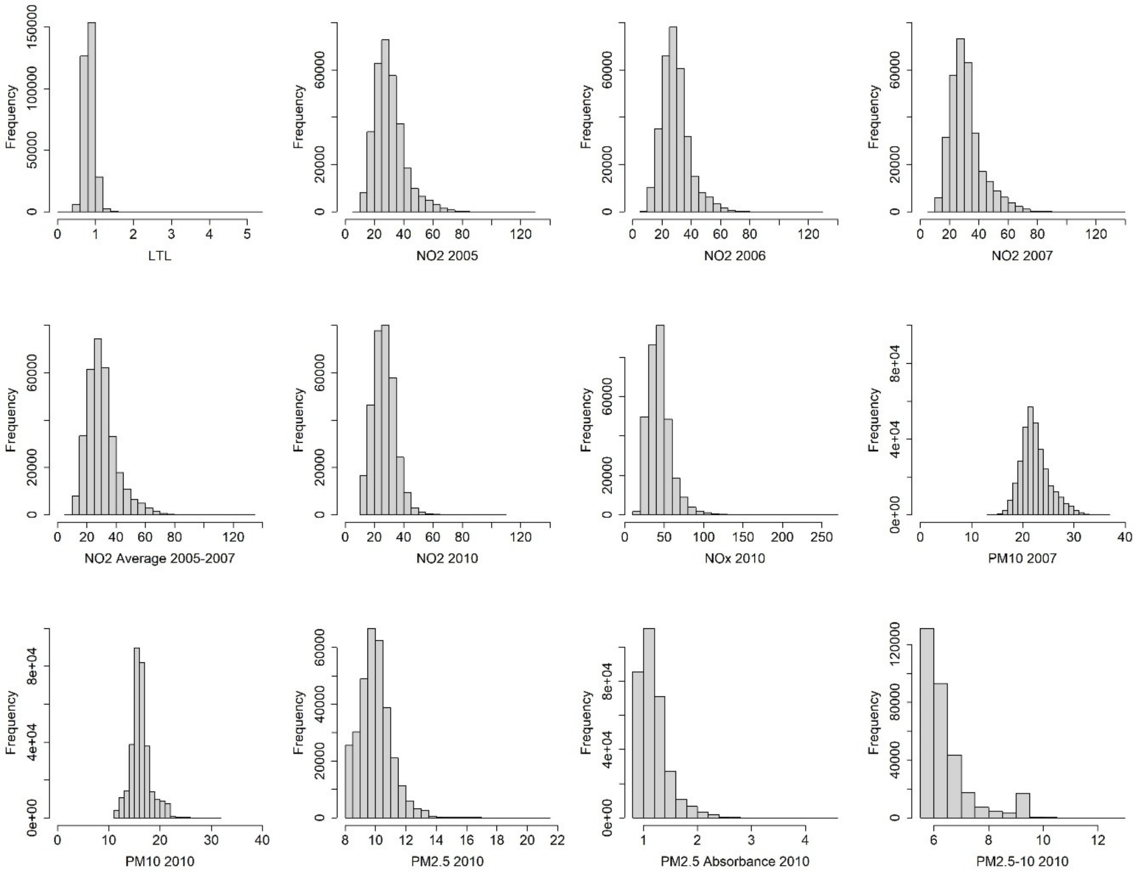

Figure S1. Histograms of leukocyte telomere length and pollutant concentrations.

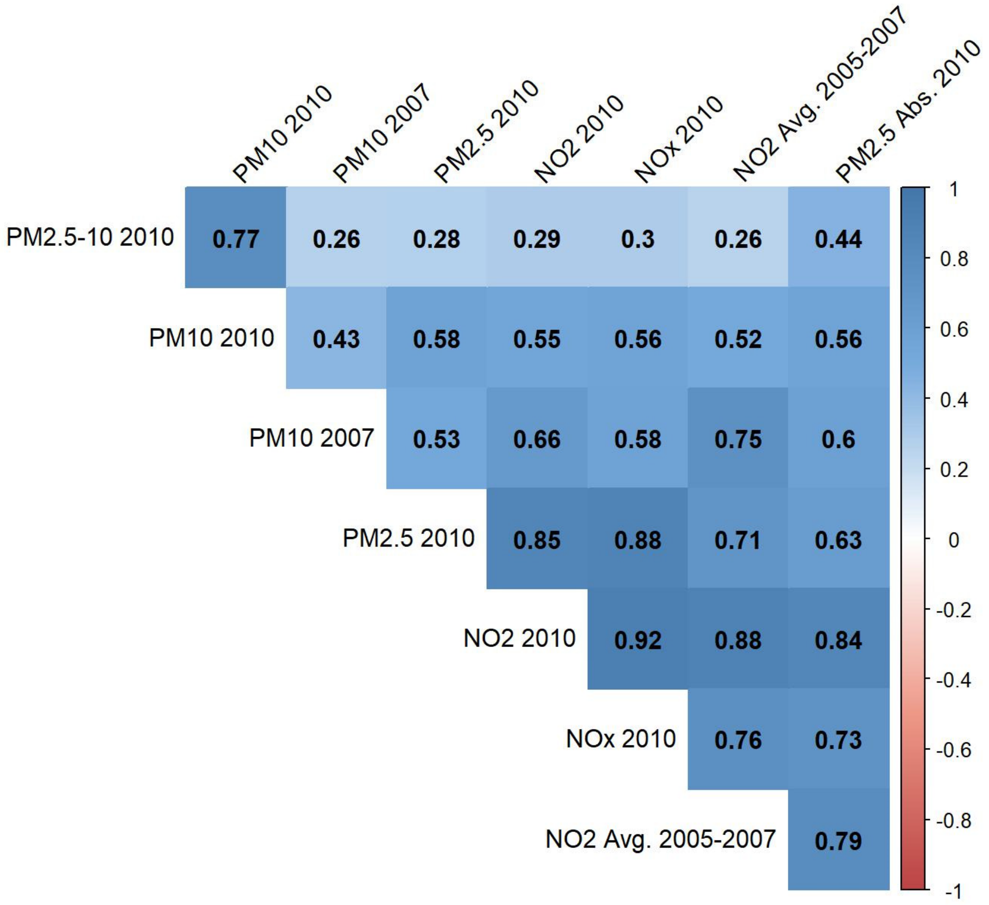

Figure S2. Spearman correlations between air pollutants.

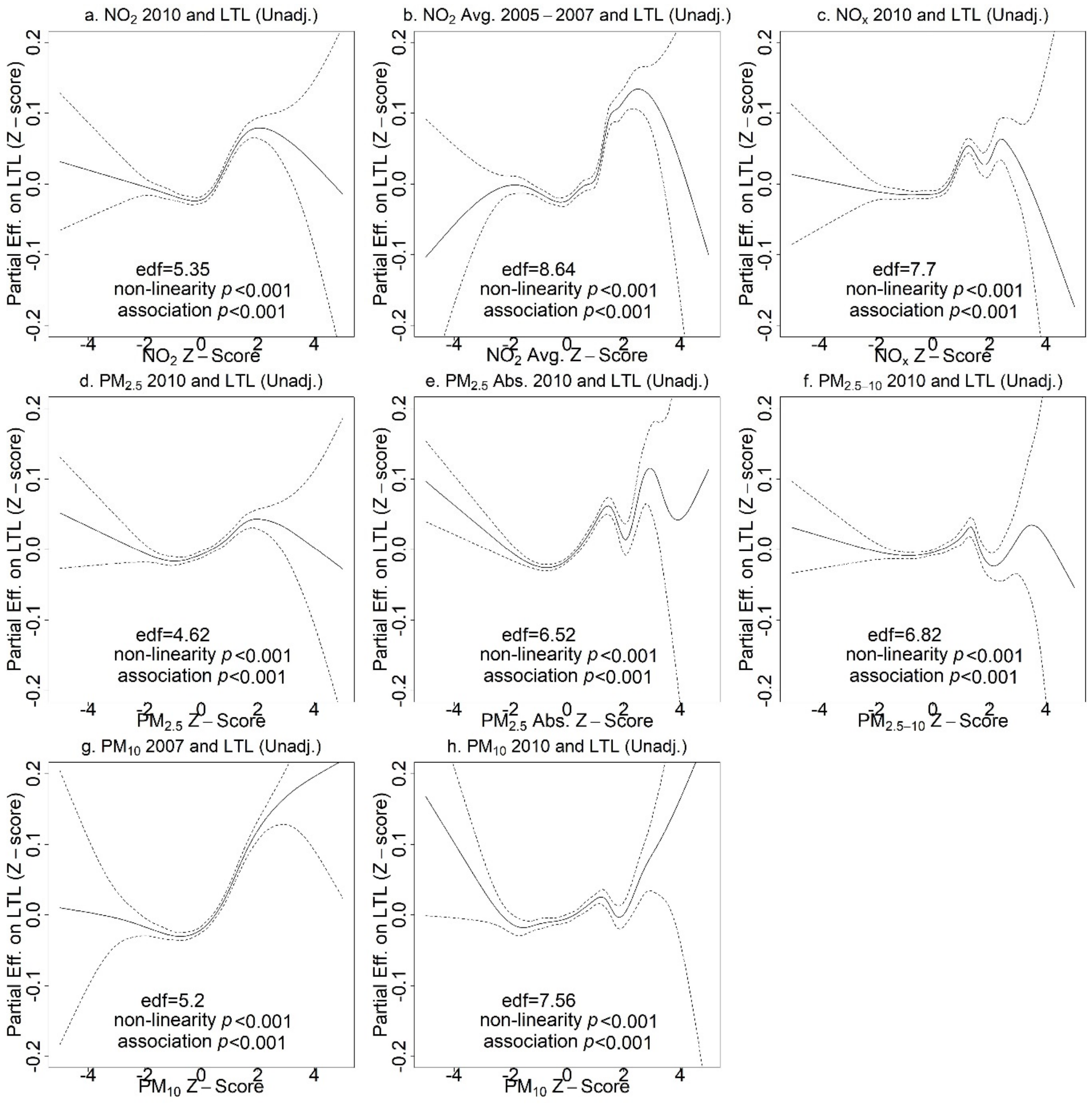

Figure S3. Generalized additive model (GAM) plots of the partial effects of air pollutants on leukocyte telomere length, with no adjustment for covariates. The tick marks on the x-axis are z-scores of an air pollutant. The y-axis represents the partial effect of each air pollutant. The areas between dash lines indicate the 95% confidence intervals. (a) NO_2 2010 and LTL; (b) NO_2 average 2005-2007 and LTL; (c) NO_x 2010 and LTL; (d) $PM_{2.5}$ 2010 and LTL; (e) $PM_{2.5}$ absorbance and LTL; (f) $PM_{2.5-10}$ 2010 and LTL; (g) PM_{10} 2007 and LTL; (h) PM_{10} 2010 and LTL.
